# Magnetization transfer imaging alterations and its diagnostic value in antipsychotic-naïve first-episode schizophrenia

**DOI:** 10.1038/s41398-022-01939-5

**Published:** 2022-05-06

**Authors:** Du Lei, Xueling Suo, Kun Qin, Walter H. L. Pinaya, Yuan Ai, Wenbin Li, Weihong Kuang, Su Lui, Graham J. Kemp, John A. Sweeney, Qiyong Gong

**Affiliations:** 1 Huaxi MR Research Center (HMRRC), Department of Radiology, West China Hospital of Sichuan University, Chengdu, 610041 Sichuan China; 2 Department of Psychiatry and Behavioral Neuroscience, University of Cincinnati, Cincinnati, OH 45227 USA; 3 Department of Biomedical Engineering, School of Biomedical Engineering & Imaging Sciences, King’s College London, London, WC2R 2LS UK; 4 Department of Psychiatry, West China Hospital of Sichuan University, Chengdu, Sichuan 610041 China; 5 Research Unit of Psychoradiology, Chinese Academy of Medical Sciences, Chengdu, Sichuan 610041 China; 6 Functional and Molecular Imaging Key Laboratory of Sichuan Province, West China Hospital of Sichuan University, Chengdu, Sichuan 610041 China; 7 Liverpool Magnetic Resonance Imaging Centre (LiMRIC) and Institute of Life Course and Medical Sciences, University of Liverpool, Liverpool, L69 3GE UK; 8 Department of Radiology, West China Xiamen Hospital of Sichuan University, Xiamen, Fujian 361022 China

**Keywords:** Schizophrenia, Neuroscience

## Abstract

Magnetization transfer imaging (MTI) may provide more sensitivity and mechanistic understanding of neuropathological changes associated with schizophrenia than volumetric MRI. This study aims to identify brain magnetization transfer ratio (MTR) changes in antipsychotic-naïve first-episode schizophrenia (FES), and to correlate MTR findings with clinical symptom severity. A total of 143 individuals with antipsychotic-naïve FES and 147 healthy controls (HCs) were included and underwent 3.0 T brain MTI between August 2005 and July 2014. Voxelwise analysis was performed to test for MTR differences with family-wise error corrections. Relationships of these differences to symptom severity were assessed using partial correlations. Exploratory analyses using a support vector machine (SVM) classifier were conducted to discriminate FES from HCs using MTR maps. Model performance was examined using a 10-fold stratified cross-validation. Compared with HCs, individuals with FES exhibited higher MTR values in left thalamus, precuneus, cuneus, and paracentral lobule, that were positively correlated with schizophrenia symptom severity [precuneus (*r* = 0.34, *P* = 0.0004), cuneus (*r* = 0.33, *P* = 0.0006) and paracentral lobule (*r* = 0.37, *P* = 0.001)]. Whole-brain MTR maps identified individuals with FES with overall accuracy 75.5% (219 of 290 individuals) based on SVM approach. In antipsychotic-naïve FES, clinically relevant biophysical abnormalities detected by MTI mainly in the left parieto-occipital regions are informative about local brain pathology, and have potential as diagnostic markers.

## Introduction

Schizophrenia, a severe psychiatric illness involving hallucinations, delusions and disorganized thinking, is one of the greatest causes of disability worldwide, affecting about 1% of the world’s population [1]. The neurobiology of this complex illness is not well understood. Neuroimaging studies have revealed distributed brain abnormalities in structure and function [2]. However, these findings have been inconsistent, potentially due to the effects of antipsychotic medication and long illness duration in many study samples [2]. Previous studies have primarily focused on volumetric and functional measures, but studying antipsychotic-naïve first-episode schizophrenia (FES) using alternative MRI approaches may be an important strategy for characterizing the neurobiological substrate and identifying useful biological markers.

Widespread structural brain abnormalities have been observed in individuals with schizophrenia. Among many reported brain abnormalities in schizophrenia, the most consistent are in the thalamo-cortical and default mode networks [2]. The macroscopic neuroimaging findings of structural brain abnormalities have been confirmed and to some extent explained by postmortem studies [3]. However, it is difficult to identify in vivo the histopathological alterations that underlie these macroscopic findings. Postmortem alterations point to changes that could be identified with MRI, but that which might be difficult to characterize with standard morphometric imaging. Techniques are needed with sufficient sensitivity to detect subtle neuropathology such as changes in the nature and concentration of macromolecules.

Magnetization transfer imaging (MTI) can provide neuropathologically relevant information in vivo unavailable by conventional T1- and T2-weighted MRI techniques [4]; this is conveniently and reproducibly characterized using the semi-quantitative magnetization transfer ratio (MTR). MTR signals are influenced by macromolecules in brain tissue, which mainly form neuronal cell membranes in gray matter and myelin in white matter [5] and is considered to be a more sensitive measure for distinguishing schizophrenia than conventional MRI measures [6–8]. Previous schizophrenia studies have found widespread MTR abnormality, e.g., in the frontal, temporal, and occipital regions [7–16]. However, the sample sizes of these prior studies were relatively small, and the majority of participants at the time of scanning were on antipsychotic medication, so it remains unclear whether the pattern of deficits reported is related to illness itself or to antipsychotic medications that are believed to impact structural alterations. Moreover, most have used region-of-interest methods which risk systematic placement bias [17–20].

Our study of a relatively large sample of individuals with antipsychotic-naïve FES used MTI to identify subtle biophysical alterations and their relation to symptom severity, and explored their potential diagnostic value. Given previous evidence of structural disruptions in schizophrenia [21, 22], we hypothesized (i) that FES would exhibit voxelwise brain MTR abnormalities and (ii) that these regional MTR alterations would be associated with symptom severity of schizophrenia. Additionally, given existing evidence that machine learning applied to structural images can identify individuals with schizophrenia [23, 24], we hypothesized that (iii) whole-brain MTR maps would distinguish individuals with FES from healthy controls (HCs) with significant classification accuracy.

## Materials and methods

### Participants

Individuals with antipsychotic-naïve FES were consecutively recruited from inpatient and outpatient programs of West China Hospital of Sichuan University from August 2005 to July 2014. The diagnosis of schizophrenia was established using the Structured Interview for the DSM-IV Axis I Disorder. Individuals with FES were recruited within days of their first hospitalization and scanned before beginning antipsychotic medication. The severity of schizophrenia symptoms was evaluated by an experienced clinical psychiatrist using the Positive and Negative Syndrome Scale (PANSS) [25]. The duration of untreated illness prior to scans was evaluated by the Nottingham Onset Schedule [26] based on information provided by the FES and their guardians and family members. We have previously reported structural MRI data from this cohort [27]: cortical thickness and surface area data from high-resolution T1-weighted MRI in 128 individuals with antipsychotic-naïve FES and 128 HCs.

HCs were recruited from the local area through poster advertisements during the same period and screened using the nonpatient edition of the Structured Interview for the DSM-IV Axis I Disorders to rule out lifetime psychiatric illness. They were from the same geographical region as the FES and had similar socioeconomic and educational backgrounds. There was no history of major psychiatric illness in their first-degree relatives. Exclusion criteria for both groups were as follows: any neurological disorder, another axis I psychiatric disorder including substance abuse or dependency, pregnancy, serious systemic illness, or MRI contraindication.

This retrospective study was approved by the Medical Research Ethics Committee of West China Hospital, Sichuan University, and written informed consent was obtained from all FES and HCs. All procedures in the present study adhered to the ethical standards of the Declaration of Helsinki and the ethical principles in the Belmont Report.

### Image acquisition

MRI of each participant was performed with the same 3.0 tesla MRI system (GE Excite) with an 8-channel phased array head coil. Whole-brain MTI scans were acquired with a three-dimensional fast low-angle shot sequence with the following parameters: repetition time, 37 ms; echo time, 5 ms; field of view, 24×24 cm^2^; flip angle, 15°; matrix size, 512×512; voxel size, 0.47×0.47×3 mm^3^; 50 contiguous axial slices; section thickness, 3 mm. One acquisition was obtained with a magnetization saturation pulse at 1.5 kHz off-resonance, then another without this pulse to produce separate images with (M_S_) and without (M_0_) saturation.

### Image preprocessing

MRIs were first reviewed by an experienced neuroradiologist to ensure that there were no gross structural abnormalities or quality flaws. Statistical Parametric Mapping software (SPM12) was used for data processing and analysis. For each patient and HC, M_S_ and M_0_ images were first co-registered using a mutual information registration algorithm. The MTR was then calculated on a voxel-by-voxel basis as MTR = 100 × (M_0_–M_S_)/M_0_, where M_0_ and M_S_ represent the signal intensities without and with the saturation pulse. As non-MTI images are partially T1-weighted, we directly normalized them to the Montreal Neurologic Institute T1-weighted template and then used the transformation parameters to normalize the coregistered MTR map. The normalized non-MTI images were skull-stripped using the brain extraction tool (http://www.fmrib.ox.ac.uk/fsl/bet/) and then applied as masks to remove nonbrain tissue in the normalized MTR maps. Finally, MTR maps were smoothed with a 6 mm full-width half-maximum 3D Gaussian kernel.

### Statistical analysis

#### Demographic and clinical data analyses

Statistical group comparisons were performed using SPSS software (IBM Corp., IBM SPSS Statistics for Windows, version 23.0). Group differences in age and education level were assessed using two-sample *t* tests, while sex differences were compared using a chi-square test. Sample size was determined using neuro power tools (http://neuropowertools.org/), which indicated that 140 participants per group would provide statistical power of 0.8.

#### Voxel-based MTR analysis

MTR maps of individuals with FES were compared with those of HCs using two-sample *t* tests in SPM12. The significance of group differences was estimated by distributional approximations from the theory of random Gaussian fields. Familywise error correction was used to correct for multiple comparisons, and clusters with at least 40 voxels with nominal *P* < 0.05 were considered significant.

#### Clinical measures association analysis

Individual mean MTR values were extracted from regions with significant intergroup differences using the volume of interest method implemented in SPM12. Partial correlation analyses were conducted between regional mean MTR values and clinical indices: PANSS Positive, Negative and Total Scores, with age, sex, and education years as covariates. Multiple comparison correction was performed using the Bonferroni correction.

#### Whole-brain MTR maps classification analysis

To assess classification effectiveness, we used whole-brain MTR maps to distinguish individuals with FES and HCs based on a support vector machine, using a C-Support Vector Classification from the python module scikit-learn (version 0.24.2; https://scikit-learn.org/) with the implementation based on libsvm. Support vector machines is the most commonly used machine learning algorithm in neuroimaging [28, 29]. By finding the hyperplane maximizing the margin between binary classes in feature space, support vector machine can learn the classification strategy from the training set, be optimized and fine-tuned with the validation set, and make individual classification decisions in a test set. Our code is available at https://github.com/QKmeans0902/MTR_classification (version 1.0).

We first applied a brain mask on the whole-brain MTR maps to exclude MTR values outside the brain, and the effects of age and sex on model performance were controlled using a Gaussian process regression model based on HCs only [30]. Then MTR values of each patient were vectorized as features for model training. Ten-fold stratified cross-validation was performed to split the training and testing sets. In this method, the participants were divided into 10 non-overlapping partitions, each with the same proportion of FES and HCs. In each one of the ten iterations of the cross-validation, 9 partitions were used as the training set, then the trained classification model was used to obtain predictions in the remaining partition. A linear kernel was used to avoid the risk of overfitting. The sole hyperparameter *C* was determined via grid search on a set of values (i.e., [10^−3^, 10^−2^, 10^−1^, 1, 10^1^, 10^2^, 10^3^]), and the grid search was performed using another nested 10-fold stratified cross-validation within the training set. Classification performance was examined based on accuracy, sensitivity, specificity and area under receiver operating characteristic curve (AUC) across 10 folds. Accuracy was determined as the percentage of correctly classified individuals among all study participants. For estimation of AUC, we plotted ROC curves showing classification performance at all classification thresholds according to true positive rate (i.e., sensitivity) and false positive rate (i.e., 1 – specificity). The AUC value was calculated as the area under the ROC curve to provide an aggregate measure of performance irrespective classification threshold selection. To estimate the significance for each ML model and data features, we performed a nonparametric permutation test to calculate a *P* value for the balanced accuracy [31]. After repeating the classification procedure 1000 times with different random permutations of the group labels, *P* is calculated as the number of times the balanced accuracy was higher for the permuted labels than the real labels, divided by 1000.

## Results

### Demographic and clinical characteristics

A total of 322 people were recruited for this retrospective study, with 18 declining to complete the study and 14 excluded based on their MTI results (Supplementary Fig. S1). Ultimately, 143 individuals with FES (mean age, 24 years ±8; 73 women; mean education years, 11.4 years ±3.5) and 147 HCs matched for age, sex, and education years (mean age, 24 years ±7; 74 women; mean education years, 11.7 years ±3.1) were included. Demographic and clinical characteristics of study participants are reported in Table 1. In individuals with FES mean illness duration was 11.1 months and PANSS Total Score was 97.6.

**Table 1:** Demographic and clinical characteristics of antipsychotic-naïve first-episode schizophrenia and healthy controls.

Characteristic	FES (*N* = 143)	HCs (*N* = 147)	*P* value
Age (years)	24 ± 8 (16–49)	24 ± 7 (16–48)	0.74
Sex (women/men)	73/70 (51.0/49.0%)	74/73 (50.3/49.7%)	0.90
Education (years)	11.4 ± 3 .5	11.7 ± 3.1	0.66
Duration of illness (months)	11.1 ± 17.9	NA	NA
PANSS scores			
Total	97.6 ± 17.8	NA	NA
Negative symptoms	19.7 ± 7.5	NA	NA
Positive symptoms	25.0 ± 6.2	NA	NA
General psychopathology symptoms	47.2 ± 9.3	NA	NA
Thought disturbance	14.2 ± 4.0	NA	NA
Activation	9.1 ± 3.4	NA	NA
Paranoid	10.1 ± 2.9	NA	NA
Depression	9.1 ± 4.3	NA	NA
Anergia	9.3 ± 4.2	NA	NA
Impulsive aggression	15.9 ± 5.2	NA	NA

Data are shown as the mean ± standard deviation except for sex, which is shown as numbers of FES and HCs (percentages). *P* < 0.05 was considered to indicate a significant difference between the groups.

*FES* first-episode schizophrenia, *HCs* healthy controls, *PANSS* Positive and Negative Syndrome Scale, *NA* Not applicable.

### Voxel-wise MTI analysis

**Fig. 1:**
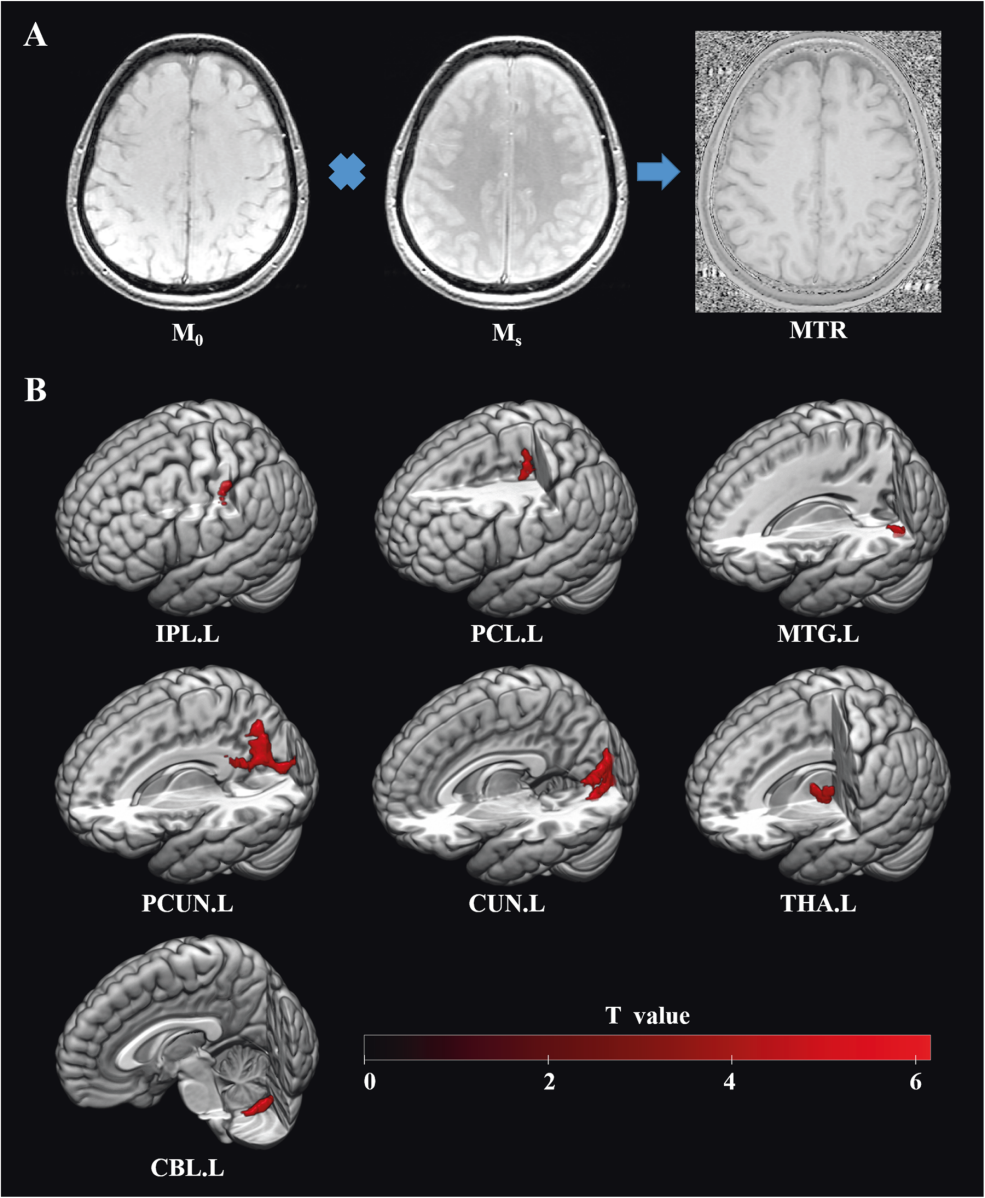
MTR in voxel-based differences between individuals with first-episode schizophrenia and healthy controls. **A** Schematic illustration of MTR calculated on a voxel-by-voxel basis as MTR = 100 × (M_0_ – M_S_)/M_0_, where M_0_ and M_S_ represent the signal intensities without and with the saturation pulse; **B** Significant clusters were identified in individuals with first-episode schizophrenia compared to healthy controls based on a voxel-level statistical threshold of *P* < 0.05 (family-wise error corrected, cluster size > 40). The color bar indicates *T* values. MTR magnetization transfer ratio, L left, CUN cuneus, PCUN precuneus, MTG middle temporal gyrus, IPL inferior parietal lobule, THA thalamus, CBL cerebellum, PCL paracentral lobule.

Compared with the HC group, the FES group exhibited higher MTR values in left hemisphere regions including the middle temporal gyrus (*P* = 0.001), inferior parietal lobule (*P* = 0.001), precuneus (*P* < 0.001), the cuneus (*P* = 0.001), paracentral lobule (*P* = 0.003), thalamus (*P* = 0.01), and cerebellum (*P* = 0.005) (familywise error corrected, cluster size > 40) (Fig. 1 and Table 2). There were no lower MTR values in FES compared to HC. Supplementary Fig. S2 shows the distribution of individual MTR values in the brain regions which differed between the FES and HC groups.

**Table 2:** Differences in magnetization transfer ratios between antipsychotic-naïve first-episode schizophrenia and healthy controls.

Predominant regions in clusters	*T* value	*P* value^a^	Cluster size	MNI coordinates (X Y Z)	MTR values FES HCs
Precuneus_L	6.16	<0.001	332	−24 −60 28	41.6 ± 2.2 39.9 ± 2.7
Inferior parietal lobule_L	5.72	0.001	43	−28 −36 40	41.1 ± 2.3 39.3 ± 2.9
Cuneus_L	5.71	0.001	270	−18 −82 12	42.4 ± 2.3 40.8 ± 2.6
Middle temporal gyrus_L	5.68	0.001	48	−42 −58 8	40.5 ± 2.3 38.7 ± 3.0
Paracentral lobe_L	5.39	0.003	50	−14 −26 56	42.6 ± 1.8 41.3 ± 2.4
Cerebellum_L	5.27	0.005	107	−28 −60 −34	36.9 ± 2.0 35.3 ± 2.9
Thalamus_L	5.12	0.010	107	−12 −10 6	44.2 ± 1.7 43.0 ± 2.3

*MNI* Montreal Neurologic Institute, *L* left, *MTR* magnetization transfer ratio, *FES* first-episode schizophrenia, *HCs* healthy controls.

^a^For peak areas of activation, multiple comparisons using the familywise error correction, with *P* < 0.05 considered a significant difference.

### Correlations between the regional mean MTR and symptom severity

**Fig. 2:**
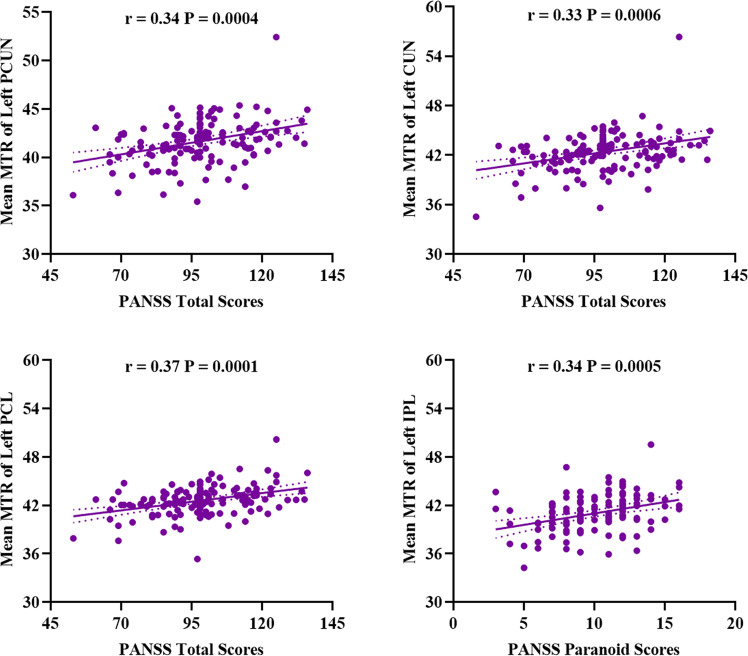
Scatter plots showing the correlations between PANSS total scores and regional mean MTR in first-episode schizophrenia. Partial correlations (presented as *r*) were conducted between regional mean MTR and PANSS score, with age, sex, and education years as covariates. Findings were significant (presented as *P*) after correction for multiple comparisons using the Bonferroni correction. MTR magnetization transfer ratio, PCUN precuneus, CUN cuneus, IPL inferior parietal lobule, PCL paracentral lobule, PANSS Positive and Negative Syndrome Scale.

Correlations were observed between PANSS Total Scores and the mean MTR within the left precuneus (*r* = 0.34, *P* = 0.0004), left cuneus (*r* = 0.33, *P* = 0.0006), and left paracentral lobule (*r* = 0.37, *P* = 0.0001), as well as PANSS Paranoid Scores and the mean MTR within the left inferior parietal lobule (*r* = 0.34, *P* = 0.0005) (Fig. 2), which all survived Bonferroni correction (*P* = 0.05/[7 MTR findings × 11 clinical characteristics] = 0.0006). There were no significant correlations between other regional MTR values and schizophrenia symptom severity (all *P* > 0.05) (Supplementary Table S1).

### Classification accuracy and performance

**Fig. 3:**
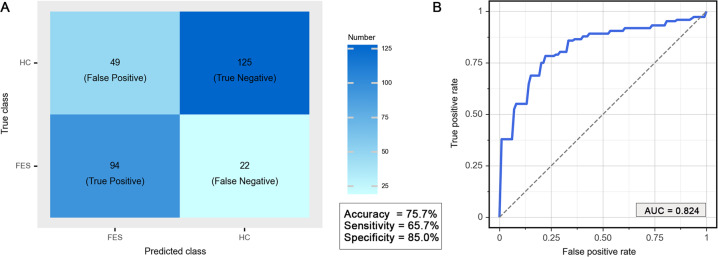
Classification performance based on whole-brain MTR maps. **A** The confusion matrix of classification between FES and HC. **B** Receiver operating characteristic curve for a prediction model based on whole-brain MTR maps using support vector machine. MTR magnetization transfer ratio, FES first-episode schizophrenia, HC healthy controls, AUC area under receiver operating characteristic curve.

Overall, the support vector machine model distinguished individuals with antipsychotic-naïve FES from HCs based on whole-brain MTR maps with an accuracy of 75.5% (219 of 290 individuals, 95% CI: 73.0%–78.0%), sensitivity 65.7% (94 of 143 FES, 95% CI: 63.0%–68.4%) and specificity 85.0% (125 of 147 HC, 95% CI: 82.9%–87.1%) (Fig. 3A, all *P* ˂ 0.05). The area under the receiver operating characteristic curve was 0.82 (95% CI: 0.80–0.85) (Fig. 3B).

## Discussion

Because medication effects and long illness duration may confound case-control MTI comparisons, we studied MTR in individuals with antipsychotic-naïve FES. We also evaluated the diagnostic value of MTR in schizophrenia. This cross-sectional study demonstrated regional microstructural MTR alterations in individuals with antipsychotic-naïve FES in left hemisphere regions including the middle temporal gyrus, inferior parietal lobule, precuneus, cuneus, paracentral lobe, thalamus, and the cerebellum. Furthermore, the regional MTR values in left precuneus and cuneus and paracentral lobule were correlated with overall clinical symptom severity. Classification models trained with MTR maps showed fair to good performance (area under the receiver operating characteristic curve 0.82).

MTR signals are influenced by brain macromolecules. A higher MTR can reflect a higher concentration of macromolecules, including myelin [32], which is important in axonal function [33]. Thus, the high MTR in these regions may suggest enhanced signal transmission in the left cerebral hemisphere that could reflect neocortical hyperexcitability related to disease or a compensation mechanism for the well-established reductions in intra- and interhemispheric connectivity in schizophrenia [34]. Of course, higher communication speed does not necessarily mean greater communication efficiency [35]. Furthermore, MTR depends not only on macromolecules but also T1 relaxation time of free water [32], so high MTR may reflect increased extracellular free water, which has been linked to inflammatory activity [36] - an important possibility, given the recent attention to neuroinflammation in schizophrenia [37]. Therefore, both myelination changes and neuroinflammation can affect the MTR signal, and future research is needed to test their contributions. Interestingly, our findings were all in the left cerebral hemisphere. This left-sided asymmetry has also been observed in diffusion tensor imaging [38]. Crow et al. [39], positing a “lateralization hypothesis of schizophrenia”, proposed that the left hemisphere may be more vulnerable to insult. This has not always been observed in MT studies but is consistent with the present findings.

Prior reports of higher MTRs confined to the uncinate and arcuate fasciculi used a tract-based region-of-interest method [34, 40, 41]. Our study investigated whole-brain differences in voxelwise analyses in FES, and found significantly elevated MTRs in multiple neocortical regions, thalamus and cerebellum, which suggests a much more widely distributed pattern of abnormalities. Some of these regions are key nodes of the default mode network [42], which is involved in self-referential processing and theory-of-mind functions [43, 44], and whose disruption has been linked to symptoms such as hallucination and paranoid ideation in schizophrenia [45]. Our findings are consistent with reports of increased neural activity of parietal [45] and occipital regions [46] as well as sensory hyperresponsiveness during acute illness [47]. The correlations between the parietal-occipital MTR alterations and PANSS scores support a critical role for microstructural disruptions of these regions in the development of symptoms. As noted above, a higher MTR could indicate greater neuronal remyelination, which might contribute to a volume increase [48]. Previous studies found a greater density of interstitial neurons in the parietal [49] and occipital areas [50] in individuals with schizophrenia, which is suggestive of neurodevelopmental abnormalities that might also contribute to the present findings.

Our FES displayed a higher MTR in the left middle temporal gyrus. fMRI studies in schizophrenia show hyperactivation in the middle temporal gyrus during auditory hallucinations [51]. However, a lower MTR in the middle temporal gyrus has been reported in medicated individuals with chronic schizophrenia [15]; this may reflect the difference between acute psychosis and persistent illness, and/or drug treatment effects. Diffusion tensor imaging studies in individuals with FES compared with HCs report lower FA in the left temporal areas [38]; this is not necessarily discrepant from our finding of higher MTR, because the two techniques measure different things: diffusion tensor imaging, responding to the orientation of water diffusion along anatomical compartments, reflects the spatial organization of fiber tracts, while MTR estimates tissue composition [14]. Future multimodal research combining both might help clarify specific white matter abnormalities in FES.

Higher MTR in the left thalamus, cerebellum and frontal regions in our FES support the findings of robust structural deficits across cerebellar-thalamo-cortical networks [2]. Previous studies showed that altered functional connectivity within prefrontal-thalamic-cerebellar circuitry is related to structural deficits [52, 53]. Our investigation extends this with novel evidence regarding the structural basis for this altered brain function.

The accuracy of case-control classification using the whole-brain MTR map was 75.5%. This is not alone sufficient for clinical application. However, MTI may have promise as part of multimodal MRI-based protocols for the identifying schizophrenia at the individual level. Although machine learning approaches are not yet ready for clinical use in psychiatry, in view of the need for objective diagnostic tests in the early stages of disease, they do offer promise for reducing misdiagnosis [28]. Improvement of classification may come from the biologically-based delineation of subgroups within the schizophrenia syndrome, and inclusion of multimodal imaging features in classification protocols [54].

Our study has limitations. First, no other imaging methods were used to verify and extend interpretation. Second, there are several ways of quantifying MTI [55]. We employed the classic MTR, used in many neuropsychiatric studies. MTR is sensitive to MRI pulse sequences and relaxation properties, making it difficult to define the specific neurobiology [48]. Emerging techniques such as the free-water imaging metric (not possible with the current imaging protocol) could provide information complementary to MTR [56]. Interpretation of our findings will require cell biology and pathology studies, and confirmation with other approaches for evaluating MT. Third, our machine learning analysis evaluated a single type of imaging modality data and one classification method. However, the models described in this paper will require replication in an independent dataset before any application in clinical decision making [57]. Finally, the cross-sectional study design limited inferences about individual changes over time and with antipsychotic treatment, which need to be examined in future longitudinal studies.

In conclusion, the present study demonstrates that MTR detects clinically relevant changes, probably reflecting macromolecular abnormalities, in the left hemisphere; these may represent an important component of the neuropathology close to the onset of schizophrenia, before antipsychotic treatment. These findings contribute to understanding the neurobiological basis of the complex clinical syndrome of schizophrenia. While not alone useful for clinical application, MTR mapping may be a useful addition to multimodal imaging profiling for diagnostic classification; this is a key aim of psychoradiology [58–61], the emerging radiological subspecialty guiding diagnosis and treatment decisions in neuropsychiatric disorders. Future research might usefully combine imaging markers as their characteristics and utility for classification are better understood, and consider different classification methods, such as Gaussian process classification and minimum spanning trees.

## Supplementary information

Supplementary Materials

## References

[1] Dhindsa RS, Goldstein DB (2016). Schizophrenia: from genetics to physiology at last. Nature.

[2] Gong Q, Lui S, Sweeney JA (2016). A selective review of cerebral abnormalities in patients with first-episode schizophrenia before and after treatment. Am J Psychiatry.

[3] Selemon LD, Goldman-Rakic PS (1999). The reduced neuropil hypothesis: a circuit based model of schizophrenia. Biol Psychiatry.

[4] Wolff SD, Balaban RS (1989). Magnetization transfer contrast (MTC) and tissue water proton relaxation in vivo. Magn Reson Med.

[5] Antosik-Biernacka A, Peuskens H, De Hert M, Peuskens J, Sunaert S, Van Hecke P (2006). Magnetization transfer imaging in chronic schizophrenia. Med Sci Monit.

[6] de Weijer AD, Neggers SF, Diederen KM, Mandl RC, Kahn RS, Hulshoff Pol HE (2013). Aberrations in the arcuate fasciculus are associated with auditory verbal hallucinations in psychotic and in non-psychotic individuals. Hum Brain Mapp.

[7] Price G, Cercignani M, Chu EM, Barnes TRE, Barker GJ, Joyce EM (2010). Brain pathology in first-episode psychosis: magnetization transfer imaging provides additional information to MRI measurements of volume loss. Neuroimage.

[8] Bagary MS, Symms MR, Barker GJ, Mutsatsa SH, Joyce EM, Ron MA, (2003). Gray and white matter brain abnormalities in first-episode schizophrenia inferred from magnetization transfer imaging. Arch Gen Psychiatry.

[9] Foong J, Maier M, Barker GJ, Brocklehurst S, Miller DH, Ron MA (2000). In vivo investigation of white matter pathology in schizophrenia with magnetisation transfer imaging. J Neurol Neurosurg Psychiatry.

[10] Foong J, Symms MR, Barker GJ, Maier M, Woermann FG, Miller DH (2001). Neuropathological abnormalities in schizophrenia: evidence from magnetization transfer imaging. Brain.

[11] Bachmann S, Haffer S, Beschoner P, Viviani R (2011). Imputation techniques for the detection of microstructural changes in schizophrenia, with an application to magnetization transfer imaging. Schizophr Res.

[12] Bohner G, Milakara D, Witthaus H, Gallinat J, Scheel M, Juckel G (2012). MTR abnormalities in subjects at ultra-high risk for schizophrenia and first-episode schizophrenic patients compared to healthy controls. Schizophr Res.

[13] Du F, Cooper AJ, Thida T, Shinn AK, Cohen BM, Ongur D (2013). Myelin and axon abnormalities in schizophrenia measured with magnetic resonance imaging techniques. Biol Psychiatry.

[14] Palaniyappan L, Al-Radaideh A, Mougin O, Gowland P, Liddle PF (2013). Combined white matter imaging suggests myelination defects in visual processing regions in schizophrenia. Neuropsychopharmacology.

[15] Faget-Agius C, Boyer L, Wirsich J, Ranjeva JP, Richieri R, Soulier E (2015). Neural substrate of quality of life in patients with schizophrenia: a magnetisation transfer imaging study. Sci Rep.

[16] Wei YB, Collin G, Mandl RCW, Cahn W, Keunen K, Schmidt R (2018). Cortical magnetization transfer abnormalities and connectome dysconnectivity in schizophrenia. Schizophr Res.

[17] Erkol C, Cohen T, Chouinard VA, Lewandowski KE, Du F, Ongur D (2020). White matter measures and cognition in schizophrenia. Front Psychiatry.

[18] Lewandowski KE, Du F, Fan XY, Chen X, Huynh P, Ongur D (2019). Role of glia in prefrontal white matter abnormalities in first episode psychosis or mania detected by diffusion tensor spectroscopy. Schizophr Res.

[19] McPhie DL, Nehme R, Ravichandran C, Babb SM, Ghosh SD, Staskus A (2018). Oligodendrocyte differentiation of induced pluripotent stem cells derived from subjects with schizophrenias implicate abnormalities in development. Transl Psychiatry.

[20] Palaniyappan L, Al-Radaideh A, Mougin O, Das T, Gowland P, Liddle PF (2019). Aberrant myelination of the cingulum and Schneiderian delusions in schizophrenia: a 7T magnetization transfer study. Psychol Med.

[21] Walterfang M, Wood SJ, Velakoulis D, Pantelis C (2006). Neuropathological, neurogenetic and neuroimaging evidence for white matter pathology in schizophrenia. Neurosci Biobehav Rev.

[22] Konrad A, Winterer G (2008). Disturbed structural connectivity in schizophrenia - primary factor in pathology or epiphenomenon?. Schizophr Bull.

[23] Xiao Y, Yan ZH, Zhao YJ, Tao B, Sun HQ, Li F (2019). Support vector machine-based classification of first episode drug-naive schizophrenia patients and healthy controls using structural MRI. Schizophr Res.

[24] Lei D, Pinaya WHL, Young J, van Amelsvoort T, Marcelis M, Donohoe G (2020). Integrating machining learning and multimodal neuroimaging to detect schizophrenia at the level of the individual. Hum Brain Mapp.

[25] Kay SR, Fiszbein A, Opler LA (1987). The positive and negative syndrome scale (PANSS) for schizophrenia. Schizophr Bull.

[26] Singh SP, Cooper JE, Fisher HL, Tarrant CJ, Lloyd T, Banjo J (2005). Determining the chronology and components of psychosis onset: The Nottingham Onset Schedule (NOS). Schizophr Res.

[27] Xiao Y, Lui S, Deng W, Yao L, Zhang W, Li S (2015). Altered cortical thickness related to clinical severity but not the untreated disease duration in schizophrenia. Schizophr Bull.

[28] Orru G, Pettersson-Yeo W, Marquand AF, Sartori G, Mechelli A (2012). Using Support Vector Machine to identify imaging biomarkers of neurological and psychiatric disease: a critical review. Neurosci Biobehav Rev.

[29] Suo XL, Lei D, Li WB, Sun HQ, Qin K, Yang J (2022). Psychoradiological abnormalities in treatment-naive noncomorbid patients with posttraumatic stress disorder. Depress Anxiety.

[30] Kostro D, Abdulkadir A, Durr A, Roos R, Leavitt BR, Johnson H (2014). Correction of inter-scanner and within-subject variance in structural MRI based automated diagnosing. Neuroimage.

[31] Golland P, Fischl B (2003). Permutation tests for classification: towards statistical significance in image-based studies. Inf Process Med Imaging.

[32] Barkovich AJ (2000). Concepts of myelin and myelination in neuroradiology. AJNR Am J Neuroradiol.

[33] Bozzali M, Wrabetz L (2004). Axonal signals and oligodendrocyte differentiation. Neurochem Res.

[34] Mandl RC, Schnack HG, Luigjes J, van den Heuvel MP, Cahn W, Kahn RS (2010). Tract-based analysis of magnetization transfer ratio and diffusion tensor imaging of the frontal and frontotemporal connections in schizophrenia. Schizophr Bull.

[35] Laughlin SB, Sejnowski TJ (2003). Communication in neuronal networks. Science.

[36] Laule C, Vavasour IM, Kolind SH, Li DKB, Traboulsee TL, Moore GRW (2007). Magnetic resonance imaging of myelin. Neurotherapeutics.

[37] Lizano P, Lutz O, Xu YX, Rubin LH, Paskowitz L, Lee AM (2021). Multivariate relationships between peripheral inflammatory marker subtypes and cognitive and brain structural measures in psychosis. Mol Psychiatry.

[38] Yao L, Lui S, Liao Y, Dua MY, Hu N, Thomas JA (2013). White matter deficits in first episode schizophrenia: an activation likelihood estimation meta-analysis. Prog Neuropsychopharmacol Biol Psychiatry.

[39] Crow TJ, Ball J, Bloom SR, Brown R, Bruton CJ, Colter N (1989). Schizophrenia as an anomaly of development of cerebral asymmetry - a postmortem study and a proposal concerning the genetic-basis of the disease. Arch Gen Psychiatry.

[40] de Weijer AD, Mandl RC, Diederen KM, Neggers SF, Kahn RS, Hulshoff Pol HE (2011). Microstructural alterations of the arcuate fasciculus in schizophrenia patients with frequent auditory verbal hallucinations. Schizophr Res.

[41] Mandl RC, Rais M, van Baal GC, van Haren NE, Cahn W, Kahn RS (2013). Altered white matter connectivity in never-medicated patients with schizophrenia. Hum Brain Mapp.

[42] Suo X, Lei D, Li N, Peng J, Chen C, Li W, et al. Brain functional network abnormalities in Parkinson’s disease with mild cognitive impairment. Cereb Cortex 10.1093/cercor/bhab520 (2022). 10.1093/cercor/bhab520 PMC9923713 35078209

[43] Buckner RL, Andrews-Hanna JR, Schacter DL (2008). The brain’s default network: anatomy, function, and relevance to disease. Ann NY Acad Sci.

[44] Romero-Garcia R, Seidlitz J, Whitaker KJ, Morgan SE, Fonagy P, Dolan RJ (2020). Schizotypy-related magnetization of cortex in healthy adolescence is colocated with expression of schizophrenia-related genes. Biol Psychiatry.

[45] Guo W, Liu F, Xiao C, Liu J, Yu M, Zhang Z (2015). Increased short-range and long-range functional connectivity in first-episode, medication-naive schizophrenia at rest. Schizophr Res.

[46] Li F, Lui S, Yao L, Hu J, Lv P, Huang X (2016). Longitudinal changes in resting-state cerebral activity in patients with first-episode schizophrenia: a 1-year follow-up functional MR imaging study. Radiology.

[47] Keedy SK, Rosen C, Khine T, Rajarethinam R, Janicak PG, Sweeney JA (2009). An fMRI study of visual attention and sensorimotor function before and after antipsychotic treatment in first-episode schizophrenia. Psychiatry Res.

[48] Jia Z, Peng W, Chen Z, Sun H, Zhang H, Kuang W (2017). Magnetization transfer imaging of treatment-resistant depression. Radiology.

[49] Kirkpatrick B, Conley RC, Kakoyannis A, Reep RL, Roberts RC (1999). Interstitial cells of the white matter in the inferior parietal cortex in schizophrenia: An unbiased cell-counting study. Synapse.

[50] Selemon LD, Rajkowska G, Goldman-Rakic PS (1995). Abnormally high neuronal density in the schizophrenic cortex. A morphometric analysis of prefrontal area 9 and occipital area 17. Arch Gen Psychiatry.

[51] Jardri R, Pouchet A, Pins D, Thomas P (2011). Cortical activations during auditory verbal hallucinations in schizophrenia: a coordinate-based meta-analysis. Am J Psychiatry.

[52] Guo WB, Liu F, Liu JR, Yu LY, Zhang J, Zhang ZK (2015). Abnormal causal connectivity by structural deficits in first-episode, drug-naive schizophrenia at rest. Schizophr Bull.

[53] Zhang X, Suo X, Yang X, Lai H, Pan N, He M (2022). Structural and functional deficits and couplings in the cortico-striato-thalamo-cerebellar circuitry in social anxiety disorder. Transl Psychiatry.

[54] Ivleva EI, Turkozer HB, Sweeney JA (2020). Imaging-based subtyping for psychiatric syndromes. Neuroimaging Clin N. Am.

[55] Sled JG (2018). Modelling and interpretation of magnetization transfer imaging in the brain. Neuroimage.

[56] Mandl RCW, Pasternak O, Cahn W, Kubicki M, Kahn RS, Shenton ME (2015). Comparing free water imaging and magnetization transfer measurements in schizophrenia. Schizophr Res.

[57] Suo X, Zuo C, Lan H, Pan N, Zhang X, Kemp GJ (2022). COVID-19 vicarious traumatization links functional connectome to general distress. Neuroimage.

[58] Gong Q, Kendrick KM, Lu L (2021). Psychoradiology: a new era for neuropsychiatric imaging. Psychoradiology.

[59] Li F, Sun H, Biswal BB, Sweeney JA, Gong Q (2021). Artificial intelligence applications in psychoradiology. Psychoradiology.

[60] Lui S, Zhou XJ, Sweeney JA, Gong QY (2016). Psychoradiology: The Frontier of Neuroimaging in Psychiatry. Radiology.

[61] Sun H, Lui S, Yao L, Deng W, Xiao Y, Zhang W (2015). Two patterns of white matter abnormalities in medication-naive patients with first-episode schizophrenia revealed by diffusion tensor imaging and cluster analysis. JAMA Psychiatry.

