# Supplementary material for: Magnetization transfer imaging alterations and its diagnostic value in antipsychotic-naïve first-episode schizophrenia
Source: Transl Psychiatry. 2022 May 6;12:189. doi: 10.1038/s41398-022-01939-5 (PMC9076920; doi:10.1038/s41398-022-01939-5)
Supplement: Supplementary file 1 — Supplementary Materials [file 41398_2022_1939_MOESM1_ESM.docx]

**Supplementary Materials**


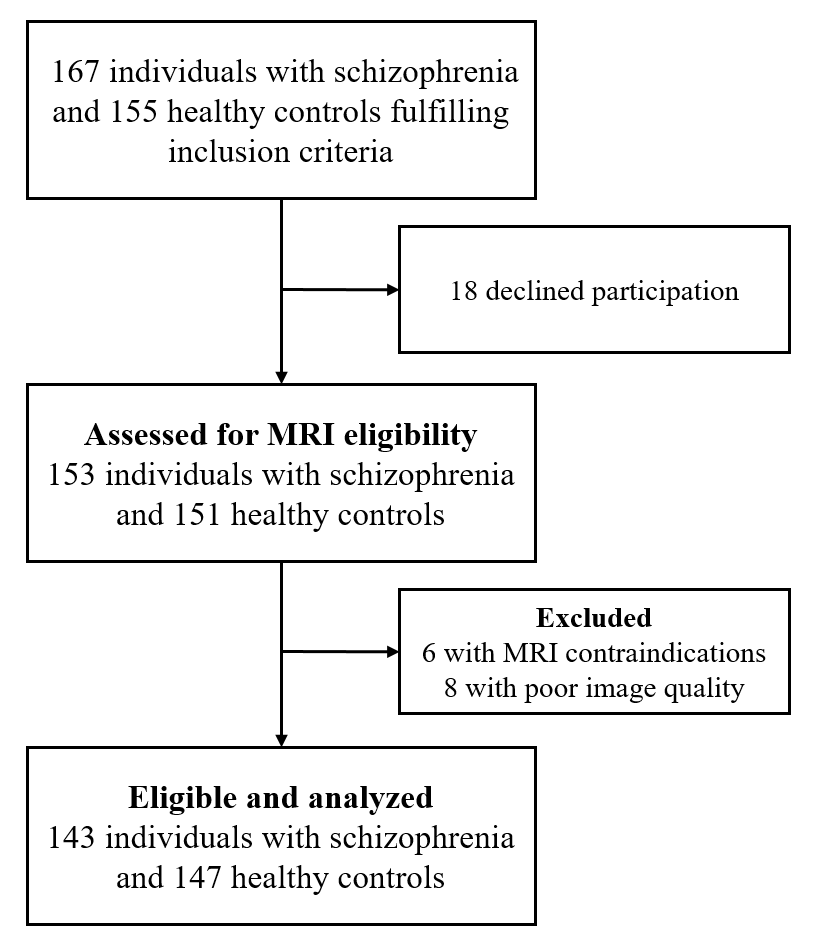


## Figure S1. Flowchart for study inclusion.

##
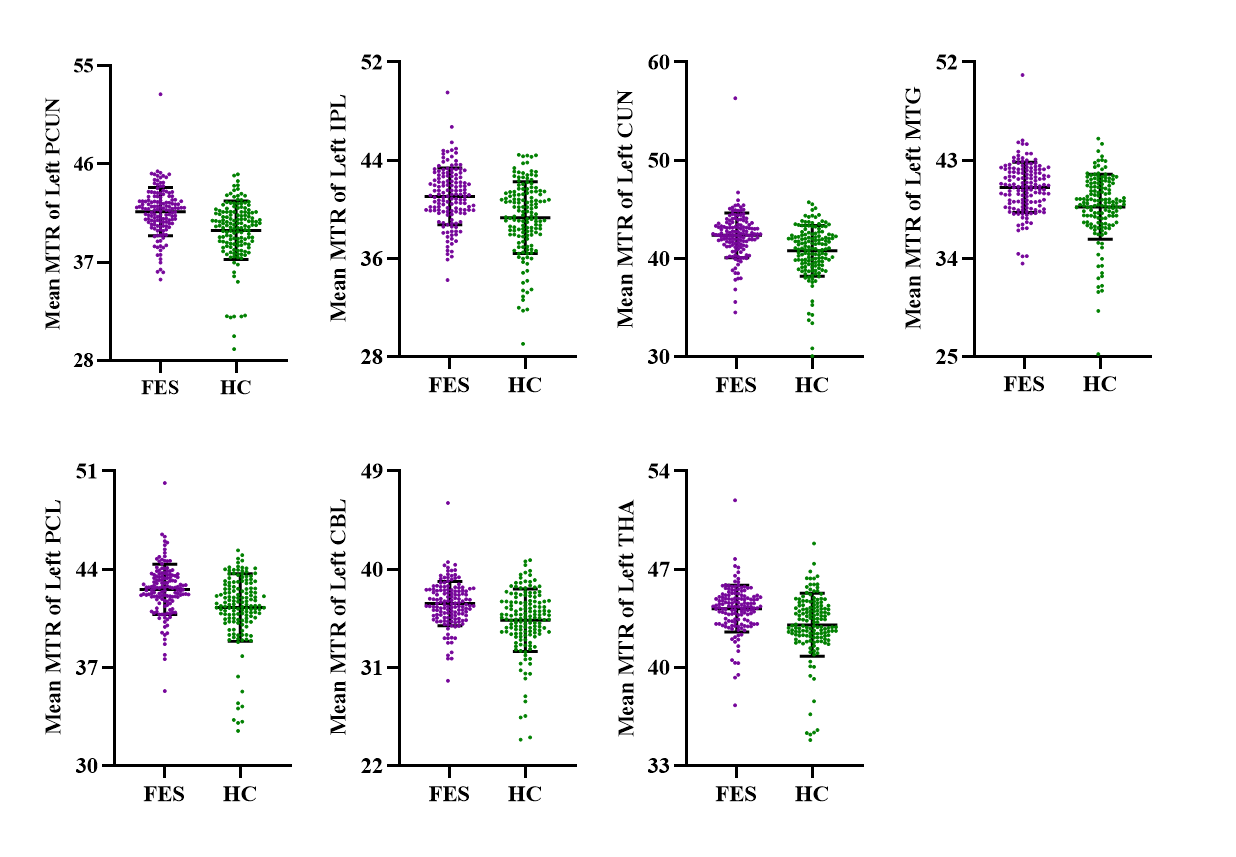
Figure S2. Distribution of the individual MTR in the altered brain regions.

MTR = magnetization transfer ratio, FES = first-episode schizophrenia, HC = healthy controls, CUN = cuneus, PCUN = precuneus, MTG = middle temporal gyrus, IPL = inferior parietal lobule, THA = thalamus, CBL = cerebellum, PCL = paracentral lobule.

**Table S1. Correlations of regional mean magnetization transfer ratios with clinical variables in antipsychotic-naïve first-episode schizophrenia**

| **﻿Area** | **PCUN L** | | **IPL L** | | **CUN L** | | **MTG L** | | **PCL L** | | **CBL L** | | **THA L** | |
| --- | --- | --- | --- | --- | --- | --- | --- | --- | --- | --- | --- | --- | --- | --- |
|  | *P* | *r* | *P* | *r* | *P* | *r* | *P* | *r* | *P* | *r* | *P* | *r* | *P* | *r* |
| **PANSS Scores** |  |  |  |  |  |  |  |  |  |  |  |  |  |  |
| Total | .0004* | .34 | .0008 | .32 | .0006* | .33 | .005 | .27 | .0001* | .37 | .02 | .23 | .002 | .30 |
| Positive symptoms | .04 | .20 | .01 | .26 | .08 | .17 | .06 | .19 | .02 | .23 | .06 | .19 | .11 | .16 |
| Negative symptoms | .02 | .24 | .17 | .14 | .03 | .21 | .004 | .28 | .02 | .23 | .27 | .11 | .17 | .14 |
| General psychopathology | .005 | .28 | .004 | .28 | .004 | .28 | .11 | .16 | .002 | .30 | .03 | .22 | .004 | .28 |
| Thought disturbance | .06 | .18 | .02 | .23 | .09 | .17 | .05 | .19 | .03 | .22 | .06 | .19 | .14 | .14 |
| Activation | .55 | .06 | .38 | .09 | .57 | .06 | .66 | .04 | .29 | .11 | .82 | -.02 | .75 | .03 |
| Paranoid | .01 | .26 | .0005* | .34 | .02 | .24 | .04 | .21 | .003 | .29 | .02 | .22 | .01 | .25 |
| Depression | .53 | .06 | .44 | .08 | .45 | .08 | .79 | -.03 | .54 | .06 | .14 | .15 | .20 | .13 |
| Anergia | .08 | .17 | .29 | .11 | .13 | .15 | .06 | .19 | .09 | .17 | .56 | .06 | .28 | .11 |
| Impulsive aggression | .10 | .16 | .06 | .18 | .23 | .12 | .33 | .10 | .16 | .14 | .19 | .13 | .06 | .19 |

The reported values are *P* and r based on partial correlations adjusting for age, sex, and education years.

* *P* < .05 was considered statistically significant (corrected for multiple comparisons using the Bonferroni correction).

Note: L = left, PCUN = precuneus, IPL = inferior parietal lobule, CUN = cuneus, MTG = middle temporal gyrus, PCL= paracentral lobe, CBL = cerebellum, THA = thalamus, PANSS = Positive and Negative Syndrome Scale.
